# Contribution of ^18^F-Fluorodeoxyglucose to the Identification of Dubious Lesions Caused by SARS-CoV-2

**DOI:** 10.3390/cimb47120984

**Published:** 2025-11-26

**Authors:** Claudia Altamura, Lorenza Marinaccio, Vincenzo Dimiccoli, Adriano Mollica, Azzurra Stefanucci

**Affiliations:** 1 Radiopharmaceutical Division, ITEL Telecomunicazioni S.r.l., 70037 Ruvo di Puglia, Italy; c.altamura@itelte.it (C.A.); v.dimiccoli@itelte.it (V.D.); 2 Department of Innovative Technologies in Medicine and Dentistry, “G. d’Annunzio” University of Chieti-Pescara, Via dei Vestini 31, 66100 Chieti, Italy; 3 Department of Pharmacy, Università degli Studi “G. d’Annunzio” Chieti-Pescara, 66100 Chieti, Italy

**Keywords:** SARS-CoV-2, Fluorodeoxyglucose (^18^F), diagnostic tools

## Abstract

Coronavirus disease, caused by the SARS-CoV-2 virus, has caused a global health crisis. While RT-PCR remains the gold standard for diagnosis, its limited sensitivity, especially in the early stages, has highlighted the need for complementary diagnostic tools. Among these, [^18^F]FDG PET/CT has gained attention for its potential role in detecting inflammation and metabolic activity associated with COVID-19. This review aims to provide an overview of current diagnostic techniques for COVID-19 and to explore the application of [^18^F]FDG PET/CT imaging in the detection and monitoring of SARS-CoV-2 infection. A comprehensive literature review was conducted on molecular, serological, and imaging-based diagnostic techniques for COVID-19, with a focus on the biological mechanism, clinical applications, and diagnostic performance of [^18^F]FDG PET/CT in COVID-19 patients. [^18^F]FDG PET/CT has demonstrated the ability to detect increased metabolic activity in COVID-19 associated pulmonary lesions, particularly ground-glass opacities, often preceding detectable morphological changes on CT. The imaging also revealed uptake in lymph nodes, bone marrow, and extrapulmonary tissues, reflecting systemic inflammation. [^18^F]FDG PET/CT represents a promising additional tool for the evaluation of inflammation and disease progression in COVID-19. However, further studies are required to define its role, optimize protocols, and assess its risk–benefit profile in the clinical setting.

## 1. Introduction

Coronavirus disease, originated by the novel SARS-CoV-2 (Severe Acute Respiratory Syndrome CoronaVirus 2) virus, started in December 2019 in the Wuhan region of China and rapidly evolved into a global pandemic, spreading throughout the world in few months [1].

Declared a Public Health Emergency of International Concern (PHEIC) by the World Health Organization (WHO) in January 2020, and subsequently classified as a pandemic in March 2020, COVID-19 has exerted unprecedented pressure on healthcare systems, public health infrastructures, and global economies [2].

The urgent need for effective diagnostic, therapeutic, and preventive strategies catalyzed one of the most accelerated scientific responses in recent history.

**Figure 1:**
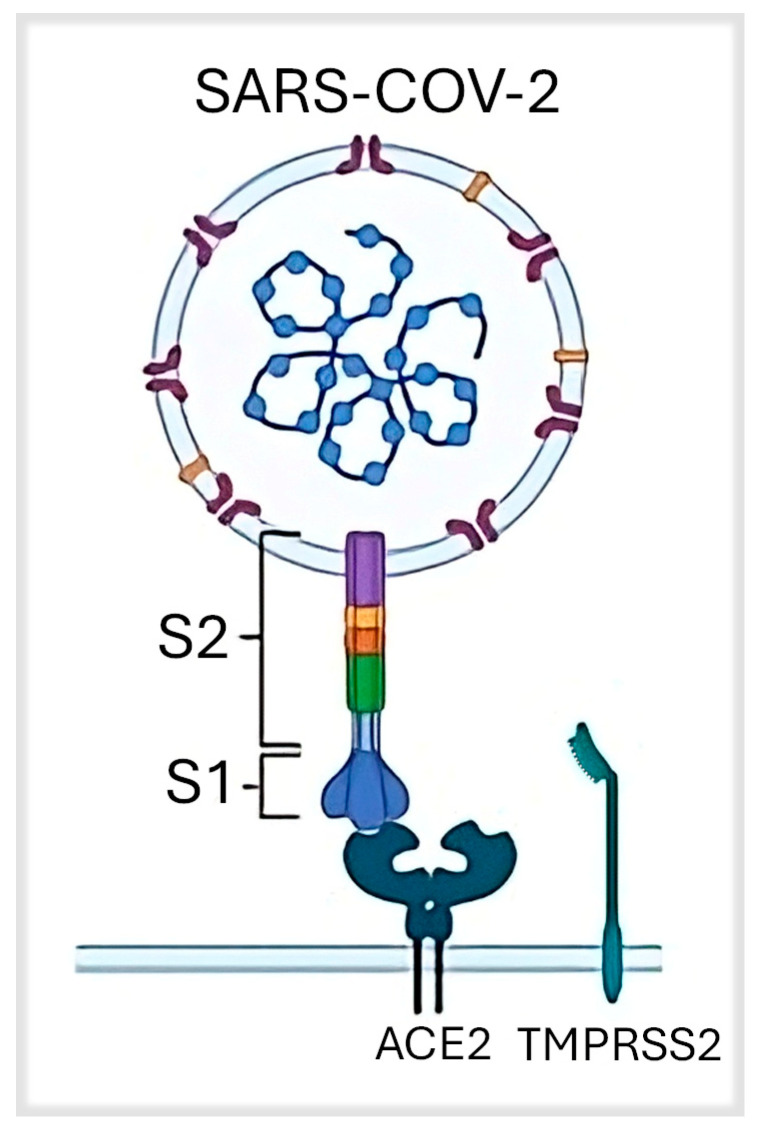
The binding between SARS-CoV-2 and spike protein [7]. Legend: ACE2, Angiotensin-converting enzyme 2; TMPRSS2, transmembrane serine protease 2; S1, Ribosomal Protein S1; S2, Ribosomal Protein S2.

SARS-CoV-2 is an enveloped, single-stranded, positive-sense RNA virus belonging to the beta-coronavirus family and can infect humans and animals. The viral genome, about 30,000 nucleotides in length, encodes essential structural proteins, such as spike (S), membrane (M), envelope (E), and nucleocapsid (N) [3]. The M protein is essential for nutrient transport, viral bud release, and envelope creation; it is a transmembrane protein. The E protein has vital importance for the assembly and release of the virus, displaying ion channel functionality [4]. N protein, the sole nucleocapsid protein, consists of two domains, both necessary for RNA binding. It also interacts with the M protein and the replicase system. The spike structure on the virus surface is composed of the trimeric S protein. The binding to the host cell receptor takes place through the S1 and S2 subunits: S1 defines the host cell spectrum, whereas S2 facilitates membrane fusion. Additionally, there are replicases and other proteins whose functions remain largely unknown [5,6]. The binding of the spike protein to the ACE2 (angiotensin-converting enzyme 2) receptor located on the plasma membrane of the host’s respiratory tract cells facilitates virus penetration (Figure 1) and consequent exploitation of the ribosomes to translate its own RNA genome, producing the proteins essential for replicating the same genetic material and assembling of new virions. Non-structural proteins are important for the transcription and replication of the viral RNA [7].

Clinically, COVID-19 infection presents with a wide range of manifestations, from asymptomatic infection to severe respiratory distress and multi-organ failure.

The most frequently reported clinical manifestations of COVID-19 infection are fever, respiratory symptoms (respiratory distress or dyspnea), rhinorrhea, tiredness, and muscle pain. In the most critical cases, SARS-CoV-2 virus causes interstitial lung disease resulting in distress syndrome and, consequently, death, especially in elderly patients and those with comorbidities. The recognition of the symptoms, along with (rt)-RT PCR (real-time reverse-transcription polymerase chain reaction), is essential for the identification of COVID-19 disease in order to isolate infected patients, initiate appropriate treatments, and prevent further transmission [8]. (rt)-RT-PCR is considered the gold-standard method for diagnosis; however, despite its high specificity, this diagnosis method has limitations in terms of sensitivity—particularly in early infection or due to sampling errors—and in terms of turnaround time [9]. In this context, radiological investigations, in particular high-resolution chest computer tomography (CT), are considered a good tool to detect and evaluate the evolution of the illness. Multifocal ground-glass opacities (GGOs), lumps, or plaques can be identified on performing a CT in the first phase of the disease; as the disease progresses, the lesion, visible on CT, may increase and occupy most of the lungs [10]. However, CT cannot be considered a specific tool for the detection of COVID-19 disease, since other pulmonary disorders reveal the same manifestations on CT.

As well as CT, several studies have been conducted on PET with Fluorodeoxyglucose (^18^F) as a useful tool for diagnosing COVID-19 infection. Fluorodeoxyglucose (^18^F) or [^18^F]FDG is an organic molecule structurally similar to glucose. It is formulated as a solution for intravenous administration. Thanks to its similarity to glucose, it enters the glucose metabolic pathway [11].

Originally developed and widely used in oncology, [^18^F]FDG PET/CT leverages the elevated glucose metabolism of pathological cells—including cancerous and inflammatory cells—to generate detailed metabolic maps of tissue activity. Its application to infectious and inflammatory diseases has gained momentum in recent years, given its ability to visualize systemic inflammation in vivo.

Given that SARS-CoV-2 infection often results in hyperinflammatory responses and that some patients exhibit extrapulmonary manifestations, [^18^F]FDG PET/CT may offer valuable insights not only for initial detection but also for monitoring disease progression and assessing systemic involvement. Additionally, the incidental detection of COVID-19-related findings in asymptomatic patients undergoing PET/CT for unrelated reasons (e.g., oncologic staging) has further stimulated interest in the potential diagnostic utility of this imaging modality.

Many studies recently focused on the potential of [^18^F]FDG PET/CT for the diagnosis of COVID-19 infections. This review aims to give a general overview of the techniques applied for the detection of COVID-19 infection and an insight into the most recent findings on the use of [^18^F]FDG PET/CT for this purpose.

Although several studies have reported incidental [^18^F]FDG PET/CT findings associated with COVID-19, a comprehensive synthesis of its diagnostic value and underlying biological rationale remains limited. This review aims to fill this gap by systematically summarizing the evidence on [^18^F]FDG PET/CT in the context of SARS-CoV-2 infection, highlighting its potential role in the identification of inflammation and disease progression. Moreover, this work provides an integrated overview of molecular and imaging-based diagnostic approaches, thereby offering a broader perspective on how [^18^F]FDG PET/CT may complement existing diagnostic modalities. By addressing both the strengths and limitations of current studies, this review contributes to defining the current state of knowledge and identifying future research priorities in this emerging field.

## 2. Techniques Applied for the Detection of COVID-19 Infection

**Figure 2:**
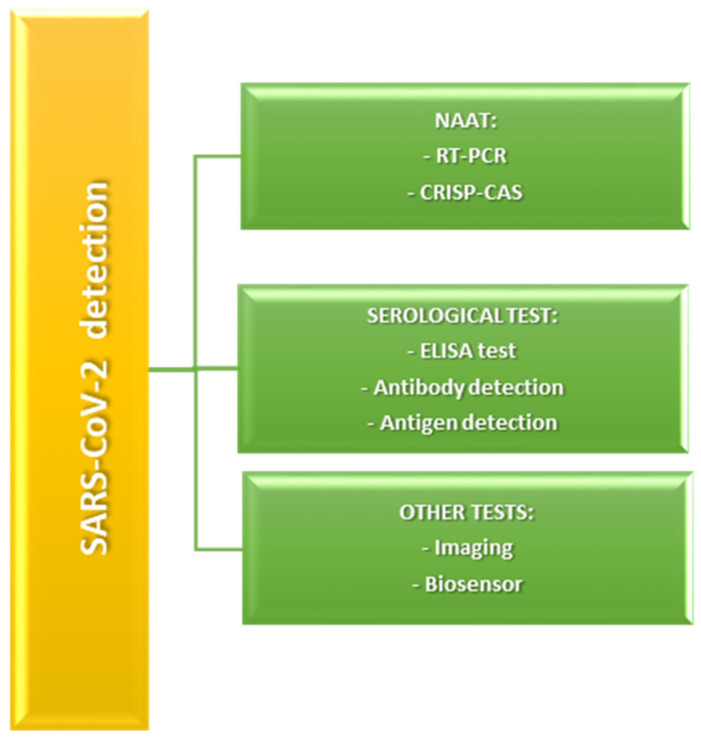
Most common SARS-CoV-2 detection methods.

Different techniques have been developed and applied for SARS-CoV-2 detection and diagnosis since the onset of the COVID-19 pandemic. These techniques range from molecular and serological assays to imaging modalities and novel biosensor technologies. A summary of the most commonly employed diagnostic methods is provided in Figure 2. The first diagnosis of COVID-19 infection is generally performed using nucleic acid amplification tests (NAATs), such as RT-PCR (reverse-transcription polymerase chain reaction) [12]. Analyses based on PCR techniques are straightforward, extremely sensitive, and precise, making them reliable tools for detecting coronavirus infections. These tests work by amplifying small amounts of deoxyribonucleic acid (DNA); the process begins with the conversion of the virus RNA into cDNA via reverse transcription. When the PCR test is carried out, the resulting DNA amplification is detected through different analytical techniques. The RT-PCR method is considered the gold standard for identifying the majority of coronaviruses. Nevertheless, this method has certain drawbacks, including the requirement for costly specialized tools and skilled technicians and specialists. Moreover, PCR tests require from 4 to 8 h to process samples, with results taking an additional 1 to 3 days, and the method has a relatively high false-negative rate [13].

In order to overcome some of the shortcomings associated with conventional RT-PCR, several NAATs have been developed. NAAT methods encompass various techniques, including recombinase-aided amplification (RPA), multiple cross-displacement amplification (MCDA), loop-mediated isothermal amplification (LAMP), nicking and extension amplification reaction (NEAR), and CRISPR–CasN-based assays utilizing Clustered Regularly Interspaced Short Palindromic Repeat (CRISPR-Cas) proteins. These methods offer the advantage of simpler reaction conditions, often not requiring thermal cycling and faster detection times, making them suitable for point-of-care (POC) applications. For instance, LAMP-based assays can yield results within 30–60 min and have been successfully integrated into portable devices for field use [14,15].

In addition to molecular methods, serological tests have emerged as essential tools, particularly for epidemiological surveillance, assessing immune response and identifying prior exposure to SARS-CoV-2. Serological tests detect specific antibodies (IgM, IgG, and IgA) or, in some cases, total immunoglobulins produced by the host in response to infection. Serological tests can be performed using methods such as lateral flow immunochromatographic strip (LFICS), lateral flow immunochromatographic assay (ILFA) [16], chemiluminescence immunoassay (CLIA) [17], and enzyme-linked immunosorbent assay (ELISA) [18]. These tests vary in their analytical performance, with ELISA and CLIA typically offering greater sensitivity and specificity compared to LFIA. However, antibody detection is generally less useful for early diagnosis, as antibodies may only become detectable several days after symptom onset. Furthermore, the persistence and neutralizing capacity of antibodies over time remain topics of ongoing investigation [19].

Antigen detection tests represent another class of diagnostic tools that provide a more direct assessment of current infection by targeting viral proteins such as the nucleocapsid (N) protein. These tests are often based on lateral flow immunoassays and offer rapid results—often within 15 to 30 min—with minimal equipment requirements. Although they are less sensitive than molecular assays, especially in asymptomatic individuals or those with low viral loads, their speed and simplicity make them valuable for mass screening and community testing efforts [20].

While molecular tests are the most widely employed technique for diagnosing COVID-19, further diagnostic methods have also been explored. In fact, beyond laboratory-based assays, imaging modalities have played a crucial role in the clinical assessment of COVID-19 disease. These consist of chest computed tomography (CT) scans along with clinical manifestation assessment [21], electrochemical (EC) biosensors [22], field-effect transistor (FET)-based biosensors [23], surface plasmon resonance (SPR)-based biosensors [24], and methods based on artificial intelligence [25]. Nonetheless, CT methods have limitations in diagnosing COVID-19, as they are unable to detect specific viruses, and numerous healthcare centers and labs do not own the required tools [26].

The ELISA method, which is based on optical measurements of fluorescent markers, has been employed for detecting SARS-CoV-2, but the process lasts several hours and requires specialized spectral analyzers [27].

Biosensors, particularly smartphone-driven ones, represent promising alternatives as diagnostic tools due to their speed, precision, and responsiveness in early identification [24]. EC biosensing assays provide various benefits, including cheapness, ease of miniaturization, and the potential for mass production. These assays also provide rapid test results and could be used in healthcare centers. However, their large-scale manufacturing is still in development, as they represent a relatively new technology [25].

Along with the distribution of vaccines and the search for new therapies, the improvement in diagnostic procedures is crucial.

In this context, different strategies have been introduced to enhance RT-PCR and biosensor-based methods. Additionally, imaging analysis, coupled with machine learning algorithms, has been extensively employed to analyze CT and X-ray images to assist in diagnosis for COVID-19 disease [28].

## 3. Discussion

Fluorodeoxyglucose (^18^F) or [^18^F]FDG is a bimolecular compound structurally very similar to glucose. Due to this similarity, it is capable of penetrating into cells through the binding with glucose transporters; it accumulates preferentially in cells using glucose as their primary energy source. [^18^F]FDG enters the metabolic glucose pathway; thus, it can be phosphorylated to FDG-6-phosphate by enzyme hexokinase. However, unlike glucose, it cannot take part in glycolysis, synthesis of glycogen, or the pentose-phosphate cycle. Moreover, [^18^F]FDG has low membrane permeability; thus, for all these reasons, it remains trapped within the cell until its dephosphorylation performed by glucose-6-phosphatase, a process that occurs slowly. Once dephosphorylated, it is primarily excreted by urine [11].

The [^18^F]FDG manufacturing process is based on the SN2 nucleophilic substitution of a leaving group in position 2 of suitably protected mannose by the radionuclide fluoride-18, with the consequent inversion of conformation and formation of the 2-deoxy-2-fluoro-D-glucose core [29]. The trifluoro methane sulfonate (triflate) group has proven to be an efficient leaving group in this synthesis. Due to the presence of fluoride-18 in position C2, [^18^F]FDG, once phosphorylated by hexokinase, cannot continue the glycolysis pathway; since the phosphorylated molecule cannot diffuse out of the cells and the dephosphorylation process takes place unhurriedly, it becomes confined and accumulates at a rate that corresponds to glucose utilization. Considering this, [^18^F]FDG accumulates at higher rates in cells with an enhanced glucose metabolism.

In inflammatory processes, activated immune cells—including neutrophils, macrophages, and lymphocytes—exhibit increased glycolytic activity, leading to enhanced [^18^F]FDG uptake. During SARS-CoV-2 infection, the hyperinflammatory response triggered by cytokine release further amplifies glucose metabolism in affected tissues. Therefore, [^18^F]FDG PET/CT provides a functional representation of inflammation and immune activation, complementing anatomical imaging modalities such as CT.

The described mechanism is the basis for using this radiopharmaceutical tracer as a tumor marker in oncology clinical practice. One of the most important characteristics of the utilization of [^18^F]FDG PET/CT is the possibility to detect the disease in the first stages, when it is very metabolically active, often before morphological changes become evident on other imaging modalities, such as CT. In fact, the first phase of disease anticipates the morphological changes in the affected area of the body, which can be identified by using other imaging tools, such as CT. Once the active phase of the disease is identified, the diagnosis and monitoring of its progression is easy to manage [29].

A substantial number of studies in the existing scientific literature have documented the incidental or unintentional identification of COVID-19-related pulmonary findings in patients who underwent [^18^F]FDG PET/CT imaging. In the majority of these cases, the PET/CT scans were originally performed for oncological evaluation, as this represents the most frequent and clinically established indication for the use of [^18^F]FDG, as a diagnostic radiotracer. Interestingly, these incidental findings were observed in individuals who were asymptomatic at the time of the examination, suggesting the potential utility of [^18^F]FDG PET/CT not only for cancer detection and staging but also for identifying unsuspected infectious or inflammatory conditions.

Comparative analysis of published studies reveals notable heterogeneity in both patient cohorts and imaging protocols. Most studies reporting incidental [^18^F]FDG PET/CT findings in COVID-19 were conducted in oncologic populations undergoing routine scans, whereas only a few prospective investigations specifically targeted infected or post-infection patients. This difference in patient selection partially explains the wide variation in reported [^18^F]FDG uptake patterns.

For instance, Setti et al. [30] and Cabrera Villegas et al. [31] observed increased pulmonary uptake predominantly in asymptomatic oncologic patients, while Dietz et al. [32] and Bahloul et al. [33] reported a stronger correlation between FDG activity and disease severity in clinically symptomatic cohorts. Similarly, differences in SUVmax values and affected lung segments suggest that inflammation intensity and disease stage influence metabolic response.

Methodological variability also contributes to inconsistent results across the literature. Some studies used low-dose CT solely for attenuation correction, whereas others employed diagnostic-quality CT, affecting lesion visibility and quantification accuracy. Time intervals between infection onset, PCR confirmation, and PET/CT acquisition further influence the degree of observed uptake.

Despite these differences, most studies converge in demonstrating increased [^18^F]FDG uptake in pulmonary ground-glass opacities and mediastinal or hilar lymph nodes, reflecting systemic inflammatory activity. However, the lack of standardized imaging protocols and quantitative thresholds limits cross-study comparability. Future investigations should therefore prioritize harmonized acquisition parameters and well-defined patient inclusion criteria to enable meta-analytical assessment of diagnostic performance.

Upon a comprehensive analysis of the reported data across various clinical studies, it appears that the proportion of asymptomatic patients undergoing [^18^F]FDG PET/CT who exhibited imaging features suggestive of COVID-19 infection is non-negligible, with estimates ranging from approximately 2% to as high as 16%. This relatively high percentage highlights the importance of maintaining clinical vigilance when interpreting PET/CT images, even in patients without respiratory symptoms or known contact with infected individuals.

In nearly all of the reported cases, the lungs are the most common sites in which the accumulation of [^18^F]FDG is evident in these patients. This observation aligns with the known pathophysiology of SARS-CoV-2, which exhibits a strong tropism for pulmonary tissue due to its interaction with angiotensin-converting enzyme 2 (ACE2) receptors, which are abundantly expressed in the respiratory epithelium. The increased metabolic activity visualized in affected lung regions is believed to reflect underlying inflammatory processes, particularly during the early or active phases of the infection, when ground-glass opacities and patchy consolidations are commonly seen on the low-dose CT component of the scan.

Therefore, the incidental detection of COVID-19-associated pulmonary abnormalities during [^18^F]FDG PET/CT imaging underscores the broader diagnostic potential of this modality and supports its utility not only in oncology but also in the identification of systemic or localized infectious processes, particularly in the context of global pandemics such as COVID-19 [30,31,32,33,34,35,36,37,38].

COVID-19 patients who undergo [^18^F]FDG-PET/CT showed heightened [^18^F]FDG intake in lung lesions, which occurs as segmental ground-glass opacities and plaques [39,40,41], frequently seen in the initial phases of the illness. SARS-CoV-2 targets cells that express ACE2 receptors and TMPRSS2 (transmembrane protease serine 2) on their exterior face. The vigorous reproduction and propagation of the virus results in pyroptosis of the host cells, triggering the release of damage-associated molecular patterns. Nearby epithelial cells, endothelial cells, and alveolar macrophages identify these patterns, leading to the stimulation of pro-inflammatory cytokines and chemokines production [41]. As a result, [^18^F]FDG intake in segmental ground-glass opacities reflects the high level of inflammation in these lesions, which is characteristic of the first phases of COVID-19 as seen on CT imaging.

In the majority of suspected COVID-19 cases identified incidentally during [^18^F]FDG PET/CT examinations, the abnormalities are primarily detected through the CT component of the scan. CT images play a crucial role in revealing characteristic pulmonary features associated with SARS-CoV-2 infection, such as ground-glass opacities, crazy paving patterns, and areas of consolidation. These radiological findings, which are commonly seen during the early or progressive stages of COVID-19 pneumonia, provide important diagnostic clues and can raise clinical suspicion even in asymptomatic individuals or those undergoing imaging for unrelated reasons.

However, it is important to note that CT performed together with PET in PET/CT analysis is typically a low-dosage scan, designed for mitigation correction and anatomical location of PET results, rather than for detailed diagnostic purposes. This low-dosage CT, which is not performed during breath-hold, has a lower diagnostic sensitivity than a dedicated chest CT and may miss smaller pulmonary lesions. Additionally, motion artifacts from free-breathing CT may produce false ground-glass opacities, especially in the inferior lung lobes [30,31,32,33,34,35,36,37,38].

A previous study investigated the diagnostic performance of CT in diagnosing COVID-19 and found that the identification of characteristic imaging features—most notably ground-glass opacities, along with other patterns associated with viral pneumonia—proved to be highly effective in supporting the diagnosis of SARS-CoV-2 infection [22]. These findings suggest that CT imaging can play a valuable role in the initial assessment, particularly in settings where rapid clinical decision-making is required or where RT-PCR testing is limited or delayed. However, despite the high sensitivity of CT in detecting pulmonary manifestations of COVID-19, it lacks sufficient specificity to be used as a standalone diagnostic tool.

Therefore, current recommendations emphasize the importance of integrating CT imaging results with molecular testing, particularly real-time reverse-transcription polymerase chain reaction (RT-PCR), which remains the gold standard for confirming the presence of SARS-CoV-2 RNA. Neither CT nor RT-PCR alone provides complete diagnostic reliability, as RT-PCR is subject to false-negative results due to factors such as sampling error or low viral load, while CT may produce false positives in cases of other viral or inflammatory lung diseases. As a result, a combined diagnostic approach utilizing both imaging and laboratory testing is strongly advised to improve overall accuracy and ensure appropriate clinical management [40].

In such circumstances, the increased [^18^F]FDG adsorption observed in areas of active inflammation may help identifying lesions caused by COVID-19 on [^18^F]FDG PET/CT scans [30,31,32,33,34,35,36,37,38].

Besides the lungs, higher [^18^F]FDG uptake in subjects with suspected COVID-19 is commonly observed in the lymph nodes, particularly in the retroperitoneal region and less often in areas outside the thoracic zone [6,14,18,20,23]. It is essential to note that the [^18^F]FDG uptake in the lymph nodes is likely due to cytokine-induced inflammation instead of direct viral infection, since lymphoid cells do not express ACE2 receptors (which are the functional entry points for SARS-CoV-2) [30,38].

Lymph node swelling is an uncommon observation in patients that undergo CT, occurring in less than 1% of patients. In some reports, the dimensions and contour of lymph nodes exhibiting significant [^18^F]FDG uptake were ambiguous; however, they are typically small, nonspecific, and have a regular shape [42].

CT scans reveal slight alterations in the dimensions of a lymph node throughout the medical course, but CT may be less effective in detecting host responses than [^18^F]FDG-PET/CT. Consequently, the true percentage of lymph node engagement might be greater than what is observed on CT. Conversely, lymph node enlargement is frequently seen in pneumonia caused by parainfluenza and adenovirus infections [42]. The uptake of [^18^F]FDG in small axillary lymph nodes is a common characteristic after the administration of the influenza vaccine [43]. Nonetheless, in COVID-19 infection, various studies reported a reduced [^18^F]FDG uptake in these lymph nodes, which could happen during the initial or less invasive phases of the illness [44]. This suggests that the immune response is minimal or almost absent in the initial phases but increases in activity as time progresses. Additionally, a decline in [^18^F]FDG uptake in lymph nodes might suggest the normalization of an excessively active body’s immune response, though further research is needed to validate this theory.

Individuals affected by COVID-19 show a variety of clinical manifestations that can involve different organs, such as the gastrointestinal system, heart, liver, bone marrow, and more [45]. Additionally, small vessel vasculitis leading to skin diseases and symptoms resembling Kawasaki disease have been observed in relation to COVID-19 [46,47]. Even so, [^18^F]FDG-PET/CT has limitations in diagnosing small or medium-sized vessel aortitis, as well as medium-to-large vessel aortitis. In cases where organ damage was determined by vasculitis, unusual [^18^F]FDG-PET/CT findings in the organs involved may indicate, in an indirect manner, the presence of small- or medium-sized vessel aortitis [48].

Endothelial tissue injury is known for being the primary reason for complications in the cardiovascular system due to COVID-19 [49]. Although [^18^F]FDG-PET/CT has not been recommended to track down FDG adsorption in the blood vessel wall, which signifies harm to the endothelium, it can depict metabolically active atherosclerosis. This occurs due to the uptake of FDG by macrophages found in atherosclerotic plaques [50,51]. Nevertheless, since COVID-19 complications often occur in elderly patients, it remains uncertain whether [^18^F]FDG can differentiate uptake caused by atherosclerosis. Further studies are needed to explore the connection among endothelial tissue injury and [^18^F]FDG adsorption in the artery wall in COVID-19.

[^18^F]FDG uptake can also highlight active thrombosis, making it a valuable tool for detecting thrombosis in COVID-19 patients. The monitoring of FDG uptake in suspected thrombosis cases may provide additional diagnostic value in these patients [52].

Increased [^18^F]FDG adsorption in bone marrow could be another imaging characteristic in patients affected by COVID-19. SARS-CoV-2 uses two kinds of receptors to penetrate in host cells: CD147 and ACE2 [53]. The receptor known as CD147 is found in mesenchymal stem cells obtained from human cord blood and bone marrow, and its presence increases in monocytes under elevated glucose levels [54,55].

This mode of operation suggests that [^18^F]FDG adsorption in the bone marrow might represent another characteristic of COVID-19. In patients with COVID-19, there is a frequent observation of elevated neutrophil counts and dispersed plasma cell infiltration in the spleen. It has been proposed that these pathological changes may result from direct viral attack and immune cell infiltration [56]. Additionally, another study using [^18^F]FDG-PET/CT imaging reported low activity in the frontal cortex of COVID-19 patients with anosmia, highlighting potential neurological involvement in these patients [57].

In order to provide a clearer overview of the available literature considered in this review, Table 1 summarizes the main [^18^F]FDG PET/CT studies investigating COVID-19-related findings, including study type, population, and major imaging results.

## 4. Conclusions and Future Directions

This review highlights the promising role of [^18^F]FDG PET/CT as an advantageous tool for monitoring inflammatory activity in patients affected by COVID-19. Given its ability to detect metabolically active inflammatory lesions, [^18^F]FDG PET/CT may offer critical insights into the systemic involvement and progression of SARS-CoV-2 infection. In selected cases—particularly in patients with confirmed COVID-19 and complex or unclear clinical presentations—it may be feasible to employ [^18^F]FDG PET/CT, provided that stringent infection control protocols and appropriate precautionary measures are rigorously followed to minimize the risk of viral transmission within the healthcare environment.

However, despite these potential advantages, current scientific evidence remains limited and insufficient to support the routine clinical use of [^18^F]FDG PET/CT in the diagnostic or management pathway of patients with suspected or confirmed COVID-19. The existing data are largely based on small cohorts, case reports, or incidental findings. Further prospective large-scale studies are essential to clarify the true diagnostic, prognostic, and monitoring value of this imaging technique in the context of COVID-19 and similar infectious diseases.

Moreover, it is important to acknowledge that, compared to conventional imaging modalities such as CT, the use of [^18^F]FDG PET/CT is inherently more complex. It requires additional logistical coordination, extended scanning times, and specialized resources, including radiotracer production and administration. These factors not only increase operational burden but also elevate the risk of exposure to infectious agents for both healthcare workers and other patients, particularly in high-throughput clinical settings. Additionally, the cumulative radiation dose from both the PET and CT components must be carefully considered, especially in vulnerable populations or in cases requiring repeated imaging.

Despite these critical limitations, the diagnostic capabilities of [^18^F]FDG PET/CT remain significant and continue to attract the attention of the research community. Its ability to provide functional and metabolic information beyond the structural details offered by conventional imaging modalities encourages ongoing investigation.

The diagnostic potential of [^18^F]FDG PET/CT is noteworthy, pushing researchers to pursue in studying it trying to reduce the limitations and increase the benefits.

Future research should aim to more precisely define the clinical role of [^18^F]FDG PET/CT in infectious diseases such as COVID-19 and similar viral outbreaks. While current evidence demonstrates its potential to visualize systemic inflammation and disease activity, several open questions remain and require systematic investigation [58,59].

Future studies should focus on the possibility of [^18^F]FDG PET/CT reliably differentiating between active viral infection and post-inflammatory or fibrotic lung lesions. This question could be addressed through longitudinal and histopathologically correlated studies, which could clarify the temporal and biological significance of PET findings in the context of viral pneumonitis.

Moreover, studies should focus on the cost-effectiveness and logistical implications of implementing [^18^F]FDG PET/CT in future pandemic scenarios. Given the high operational complexity and limited availability of radiopharmaceuticals, health-economic evaluations and workflow optimization models are needed to assess whether PET/CT could serve as a targeted, rather than routine, diagnostic resource in infectious disease management [60,61,62].

Multicenter collaborations should focus on harmonizing acquisition parameters, disinfection protocols, and patient-handling workflows to minimize infection risk without compromising image quality.

Finally, future studies comparing [^18^F]FDG with novel, infection-specific tracers (e.g., targeting immune cell activation or viral replication pathways) may broaden the diagnostic scope of molecular imaging in infectious diseases.

Overall, addressing these research directions will help establish evidence-based guidelines for the safe, effective, and economically sustainable use of PET/CT in pandemic preparedness and management.

## Figures and Tables

**Table 1:** Summary of key [^18^F]FDG PET/CT studies in COVID-19.

Authors (Year)	Study Type	Patient Population/Sample Size	Main PET/CT Findings	Key Conclusions
Setti et al., 2021 [30]	Retrospective, oncologic patients	65 asymptomatic cancer patients	Incidental interstitial pneumonia detected on PET/CT during COVID-19 pandemic; increased [^18^F]FDG uptake in GGOs.	PET/CT may incidentally detect COVID-19 in asymptomatic individuals.
Cabrera-Villegas et al., 2021 [31]	Retrospective	12 oncologic patients	FDG uptake in bilateral pulmonary GGOs, compatible with viral pneumonia.	PET/CT can reveal unexpected COVID-19-related findings in cancer patients.
Dietz et al., 2021 [32]	Prospective observational	30 hospitalized COVID-19 patients	Correlation between whole-body inflammatory activity and disease severity.	Higher [^18^F]FDG uptake associated with poorer short-term outcomes.
Bahloul et al., 2021 [33]	Observational	20 confirmed COVID-19 patients	Lower pulmonary FDG uptake in confirmed COVID-19 cases than expected.	Suggests variability in inflammatory response; PET/CT role may depend on disease stage.
Halsey et al., 2021 [35]	Retrospective	Mixed (oncologic + inflammatory cases)	Incidental FDG uptake in lungs, lymph nodes in asymptomatic COVID-19 cases.	PET/CT may detect subclinical inflammation before symptoms.
Olivari et al., 2020 [38]	Case series	4 patients	Bilateral GGOs with increased [^18^F]FDG uptake.	Early pulmonary metabolic changes visible before severe symptoms.
Mucientes Rasilla et al., 2020 [37]	Case report	1 oncologic patient	Incidental pneumonia with FDG-avid GGOs.	PET/CT can contribute to early identification of COVID-19 lesions.
Maurea et al., 2020 [36]	Retrospective, multicentric	10 PET/CT centers in Italy	FDG uptake in lungs and lymph nodes in COVID-19-positive patients.	Confirms systemic inflammatory involvement detectable via PET/CT.

## Data Availability

No new data were created or analyzed in this study. Data sharing is not applicable to this article.

## Abbreviations

The following abbreviations are used in this manuscript:

SARS-CoV-2	Severe Acute Respiratory Syndrome COronaVirus 2
RT-PCR	Reverse Transcription Polymerase Chain Reaction
[^18^F]FDG	FluoroDeoxyGlucose (^18^F)
PET	Positron Emission Tomography
CT	Computed Tomography
COVID-19	COronaVIrus Disease 19
RNA	RiboNucleic Acid
S	Spike protein
M	Membrane protein
E	Envelope protein
N	Nucleocapsid protein
ACE2	Angiotensin-Converting Enzyme 2
(rt)-RT PCR	Reverse Transcription—Real Time Polymerase Chain Reaction
GGOs	Ground-Glass Opacities
NAATs	Nucleic Acid Amplification Tests
DNA	DeoxyriboNucleic Acid
cDNA	Complementary DNA
RPA	Recombinase-Aided Amplification
MCDA	Multiple Cross Displacement Amplification
LAMP	Loop-Mediated Isothermal Amplification
NEAR	Nicking and Extension Amplification Reaction
CRISPR	Clustered Regularly Interspaced Short Palindromic Repeat
LFICS	Lateral Flow ImmunoChromatographic Strip
ILFA	Lateral Flow Immunochromatographic Assay
CLIA	ChemiLuminescence ImmunoAssay
ELISA	Enzyme-Linked ImmunoSorbent Assay
EC	ElectroChemical
FET	Field-Effect Transistor
SPR	Surface Plasmon Resonance
SN2	Substitution Nucleophilic Bimolecular
TMPRSS2	TransMembrane PRoteaSe Serine 2
